# The microbiomic signature of hemorrhoids and comparison with associated microbiomes

**DOI:** 10.3389/fmicb.2024.1329976

**Published:** 2024-05-13

**Authors:** Yuquan Wang, Wenya Su, Zhiqiang Liu, Yihua Wang, Ling Li, Hai Xu, Mingyu Wang, Wenlong Shen

**Affiliations:** ^1^Department of Anorectal Surgery, Qilu Hospital of Shandong University (Qingdao), Shandong, China; ^2^State Key Laboratory of Microbial Technology, Microbial Technology Institute, Shandong University, Jinan, Qingdao, China

**Keywords:** hemorrhoids, microbial community structure, bacterial infection, microbiomic, thrombosed hemorrhoid

## Abstract

Hemorrhoids are a common ailment that can cause significant disruptions to one’s daily life. While some researchers have speculated about a potential link between hemorrhoid development and gut microbes, there is currently insufficient evidence to support this claim. In this study, we collected samples from 60 hemorrhoid patients and analyzed the composition and characteristics of microbiomes in hemorrhoids. PCoA results revealed distinct differences between the microbiomes of hemorrhoids, skin-originated microbiomes, and gut microbes, highlighting the complex nature of hemorrhoidal microbiomes. The distribution characteristics of *Staphylococcus* suggest that the skin microbiome influences the microbiome of hemorrhoids. Additionally, we observed higher levels of *Prevotella* in two cases of thrombosed hemorrhoids compared to non-thrombosed hemorrhoids. This finding suggests that *Prevotella* may play a crucial role in the development of thrombosed hemorrhoids.

## Introduction

Hemorrhoids are the most common condition in the field of proctology, with a prevalence ranging from 4 to 38.93% (Johanson and Sonnenberg, 1990; Riss et al., 2012). This condition typically involves the displacement of the anal cushion, leading to discomfort and impacting the daily lives of those affected (Loder et al., 1994). Hemorrhoids can be classified into internal hemorrhoids and external hemorrhoids based on their location and clinical manifestations (Adler, 1905). In addition, thrombosed hemorrhoids are a specific type of hemorrhoids characterized by the formation of blood clots within the hemorrhoidal blood vessels. This condition results in swelling, redness, and pain in the hemorrhoidal tissues (Zhang et al., 2019).

In the medical field, there is no consensus on the etiology of hemorrhoids. Currently, there are several main theories on the causes of hemorrhoids: (1) the varicose vein theory, (2) the vascular hyperplasia theory, and (3) the theory of sliding anal canal lining, which is widely accepted (Palumbo et al., 2023). The earliest theory proposed that hemorrhoids may be related to varicose veins, such as varicose swelling caused by increased local pressure from hard stools (Burkitt, 1972; Chawla and Dilawari, 1989). Some scholars believe that the formation of hemorrhoids may be a result of vascular proliferation during the inflammatory response and tissue remodeling process (Chung et al., 2004; Aigner et al., 2009). The latest theory suggests that the pathological sliding of the anal cushions is the main cause of hemorrhoid formation (Thomson, 1975; Haas et al., 1984; Loder et al., 1994; Lohsiriwat, 2012). The above three theories are based on the characteristics of human tissues. In addition to the reasons mentioned above, dietary habits such as a low-fiber diet, lack of regular exercise, and low water intake are also believed to be associated with the formation of hemorrhoids (Feyen et al., 2022).

In the past decade, with the rapid advancement of microbiology, people have gradually realized that the role of microorganisms is far more powerful than we imagined. The traditional concept of the gut-brain axis has also gradually transformed into the gut-brain-microbiome (GBM) interactions. Many researchers believe that there is a close relationship between microorganisms and the development of hemorrhoids, particularly the microorganisms residing in the gut. Current research shows that the intestinal microbiota plays a crucial role in the development of the host immune system, digestion, and other pathways. Moreover, an increasing number of studies have demonstrated a correlation between fecal microbiota and chronic constipation (Jeffery et al., 2012; Parthasarathy et al., 2016).

With the popularity of high-throughput sequencing technology, the 16S ribosomal RNA (rRNA) gene amplicon sequencing has also been widely used in various gastrointestinal diseases (Takahashi and Andoh, 2016; Nishino et al., 2018). Compared with traditional microbiological culture, sequencing technology can accurately and realistically reflect the composition and proportion of microbial communities with high throughput (Lundberg et al., 2013). Furthermore, based on the viewpoint that the majority of microorganisms in different environments are unculturable, 16S rRNA can accurately represent the microbial composition in a given environment (Steen et al., 2019). From the above considerations, in the study on the correlation between the clinical occurrence of hemorrhoids and microbiota, in order to find the relationship between microbe and hemorrhoids, 16S rDNA amplicons sequencing was used to analyze the samples of 60 patients with hemorrhoids.

## Materials and methods

### Sample collection and preparation

The samples were taken from various regions of patients with hemorrhoids in the anorectal department of Qilu Hospital of Shandong University (Qingdao). When sampling, use a sterile cotton swab along with an anal reamer to carefully collect an adequate amount of tissue fluid from the relevant sites. The swabs are then quickly placed in sterile Eppendorf tubes containing 15% glycerin, stored at a low temperature, and transported to the lab.

### 16S rDNA amplicon sequencing and bioinformatics analysis

Total DNA was extracted from swab samples using the DNeasy® PowerSoil® Kit (Qiagen, Germany). The extracted DNA was checked using the NanoDrop One spectrophotometer (NanoDrop Technologies, Wilmington, DE) and the Qubit 3.0 Fluorometer (Life Technologies, Carlsbad, CA, United States). Amplification of the 16S V3-V4 region was performed using a custom primer with a barcode (341F: 5′-CCTACGGGNGGCWGCAG-3′, 805R: 5′-GACTACHVGGGTATCTAATCC-3′). Utilizing the library building kit from Vazyme, specifically the VAHTS® Universal DNA Library Prep Kit for Illumina. 16S rDNA amplicon sequencing was performed on the Illumina NovaSeq 6000 sequencer (Illumina, Inc., San Diego, CA, USA). Using QIIME2 v2021.4.0, the primer sequences were removed (Bokulich et al., 2018). Using the QIIME2 plugin vsearch (with a default sequence threshold of 97%), Operational Taxonomic Units (OTUs) classification was determined. The SILVA ribosomal RNA database (v138.1) was used for annotation.

### Data analysis and statistics

Unweighted UniFrac distance was calculated using the phyloseq package in the R platform. Hierarchical clustering was performed using the UPGMA method. MaAsLin2 (Microbiome Multivariable Association with Linear Models version 2.0) was used to identify biomarkers of microbiomes using linear models. ANOSIM and NMDS were performed using the vegan package on the R platform.

All statistical analyses were performed using Jamovi 2.3.21.0 or Prism 9.4.1 (681).

## Results and discussion

### Sample collection and experimental design

Samples were collected from 60 hemorrhoid-suffering patients to investigate the microbiomic signatures of hemorrhoids and the potential origins of microbes associated with hemorrhoids. These patients include 22 males and 38 females, with an average age of 43.75. Standard diagnostic procedures were performed to confirm the disease, and samples were taken before and after surgery (for hemorrhoids).

**Figure 1:**
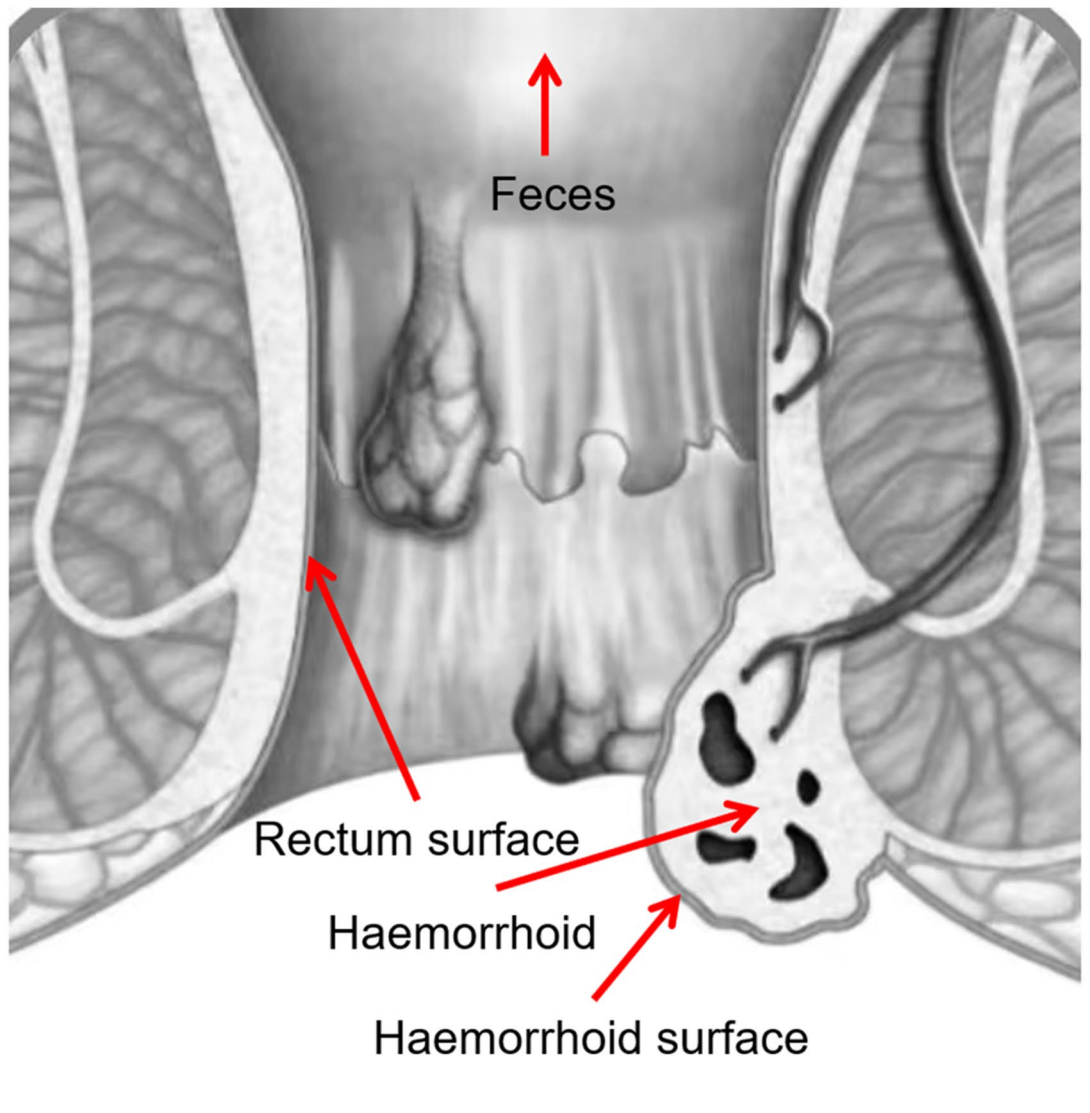
Sampling locations.

Four samples were taken from each patient, including swabs of feces, rectum surface, hemorrhoid surface before surgery, and hemorrhoid after surgery (Figure 1). These sampling sites were designed to identify potential microbiomic biomarkers for each site, especially for hemorrhoids, and to evaluate the potential origins of the hemorrhoidal microbes.

### Bacterial compositions of collected samples

**Figure 2:**
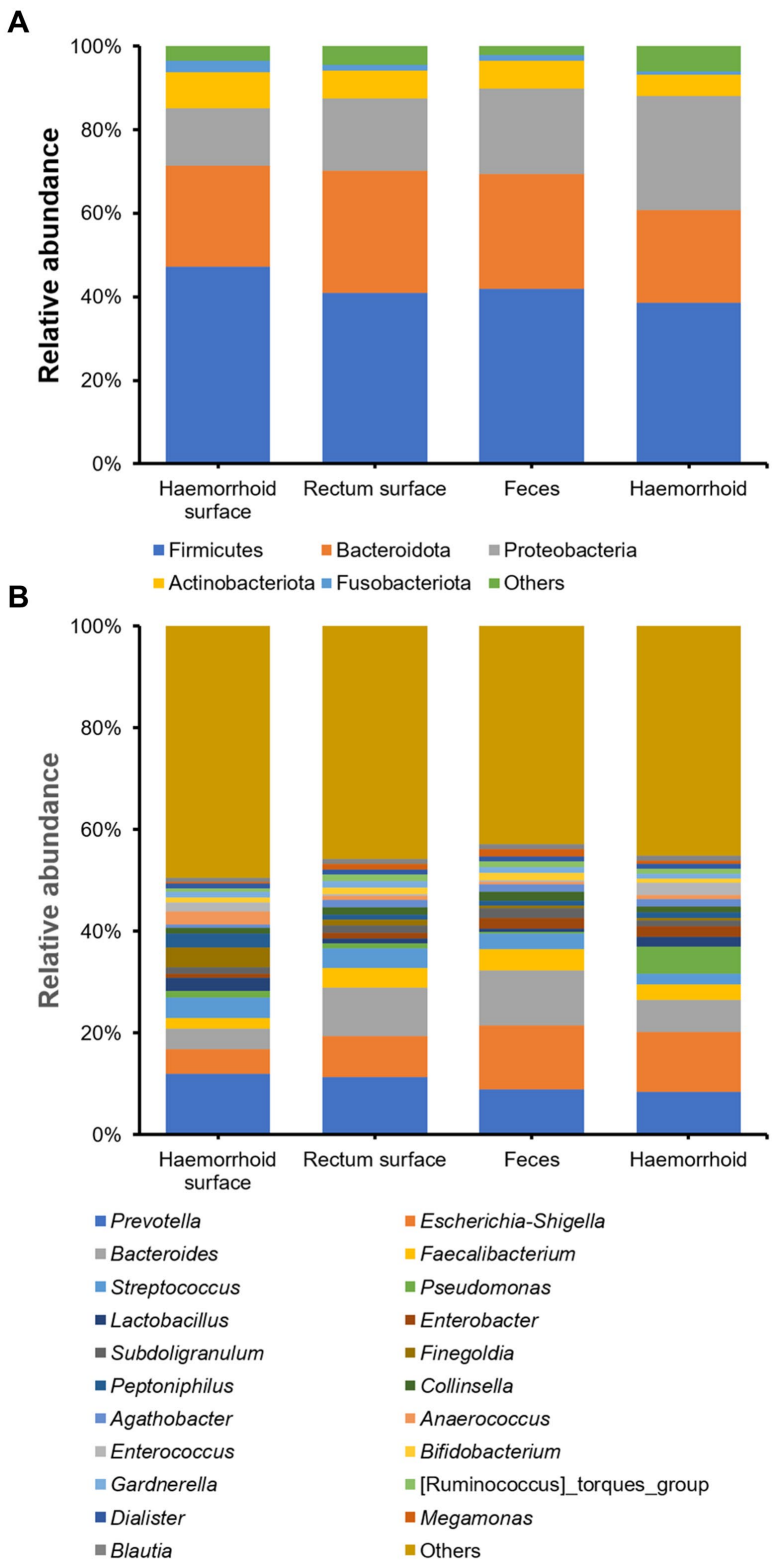
Microbial community structures of the four sampling locations. **(A)**, on phylum level; **(B)**, on genus level.

The bacterial compositions of the collected samples were determined using 16S rDNA amplicon sequencing. On the phylum level, Firmicutes, Proteobacteria, and Bacteroidota are the three most abundant bacterial taxa, consistent with prior research on human gastrointestinal microbiomes (Figure 2A). On the genus level, *Prevotella*, *Escherichia-Shigella*, *Bacteroides*, *Faecalibacterium*, and *Streptococcus*, are present at high abundances. These genera are also frequently observed in gastrointestinal tract samples (Figure 2B).

**Figure 3:**
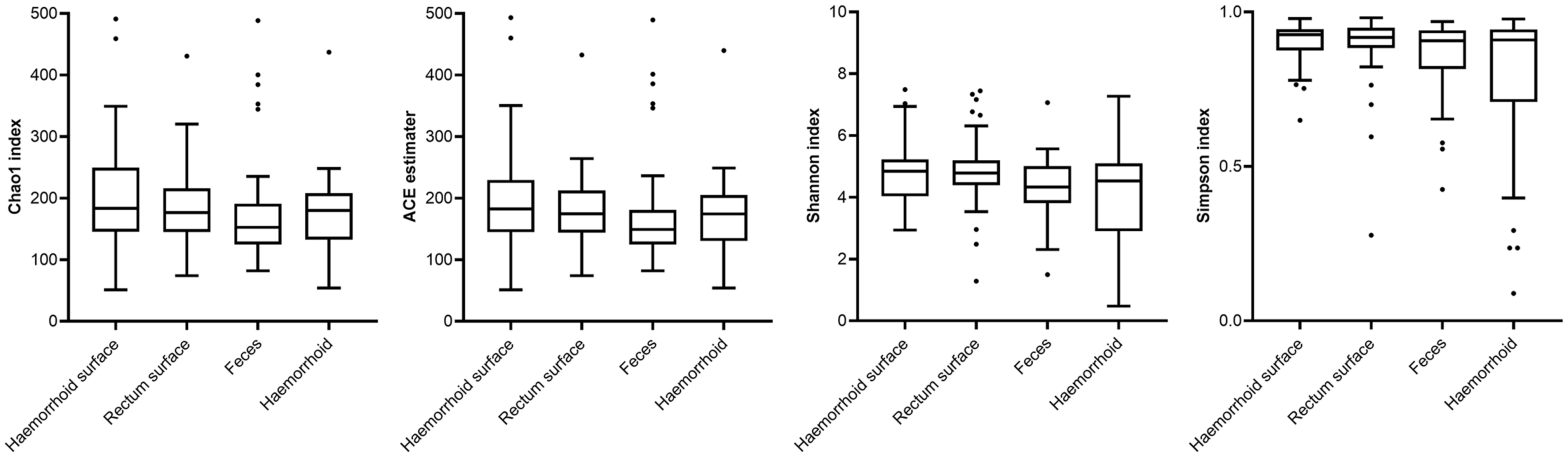
Comparison of α-indices.

Analysis of alpha diversities revealed that the microbiomes of the hemorrhoid surface and rectum surface exhibit similar alpha diversities (Figure 3; Table 1). Similarly, feces and hemorrhoid microbiomes exhibit similar α diversities (Figure 3; Table 1). However, both feces and hemorrhoid microbiomes exhibit lower richness (as indicated by Chao1 indices and ACE estimators) and diversity (as measured by Shannon indices and Simpson indices) levels. Therefore, feces and hemorrhoid carry microbiomes with lower diversities, which can be explained by the lack of exposure of hemorrhoid and feces to the environment. This is particularly evident when considering that the skin has been removed from the hemorrhoids. However, it is surprising to discover that hemorrhoid microbiomes are as diverse as fecal microbiomes, which could be one of the most diverse bacterial communities inside the human body. This reveals the complexity of microbes in hemorrhoids.

**Table 1:** Statistical significance of comparison between alpha indices.

Sample	Alpha indices	Hemorrhoid surface	Rectum surface	Feces	Hemorrhoid
Hemorrhoid surface	Chao1 index	n.a.	n.s.	**	*
	ACE estimater	n.a.	n.s.	*	*
	Shannon index	n.a.	n.s.	**	*
	Simpson index	n.a.	n.s.	*	**
Rectum surface	Chao1 index	n.s.	n.a.	**	*
	ACE estimater	n.s.	n.a.	**	*
	Shannon index	n.s.	n.a.	***	***
	Simpson index	n.s.	n.a.	*	***
Feces	Chao1 index	**	**	n.a.	n.s.
	ACE estimater	*	**	n.a.	n.s.
	Shannon index	**	***	n.a.	n.s.
	Simpson index	*	*	n.a.	*
Hemorrhoid	Chao1 index	*	*	n.s.	n.a.
	ACE estimater	*	*	n.s.	n.a.
	Shannon index	*	***	n.s.	n.a.
	Simpson index	**	***	*	n.a.

n.a., Not available; n.s., Not significant; ^*^p < 0.05; ^**^p < 0.01; ^***^p < 0.001.

**Figure 4:**
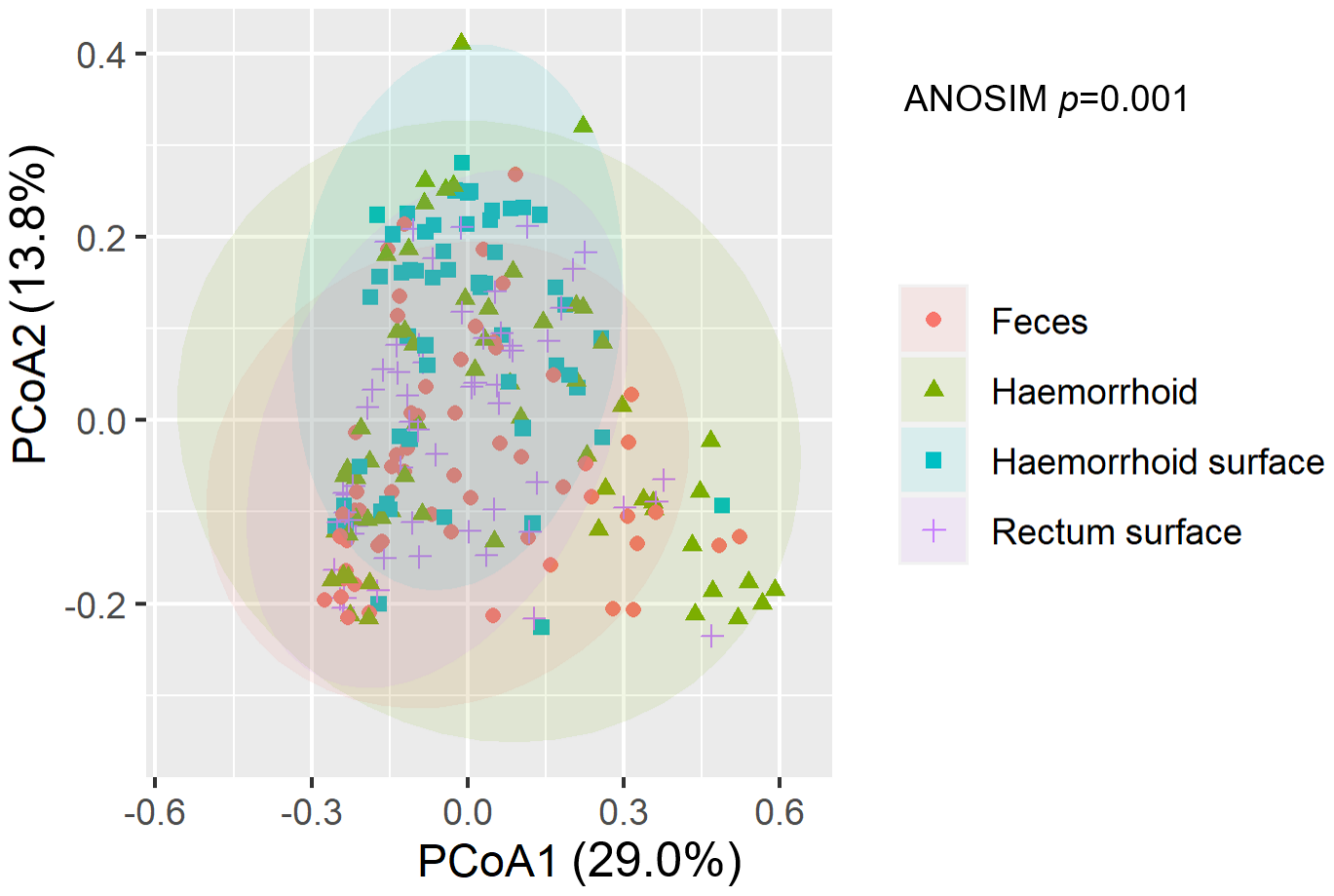
NMDS analysis of the microbiomes.

**Figure 5:**
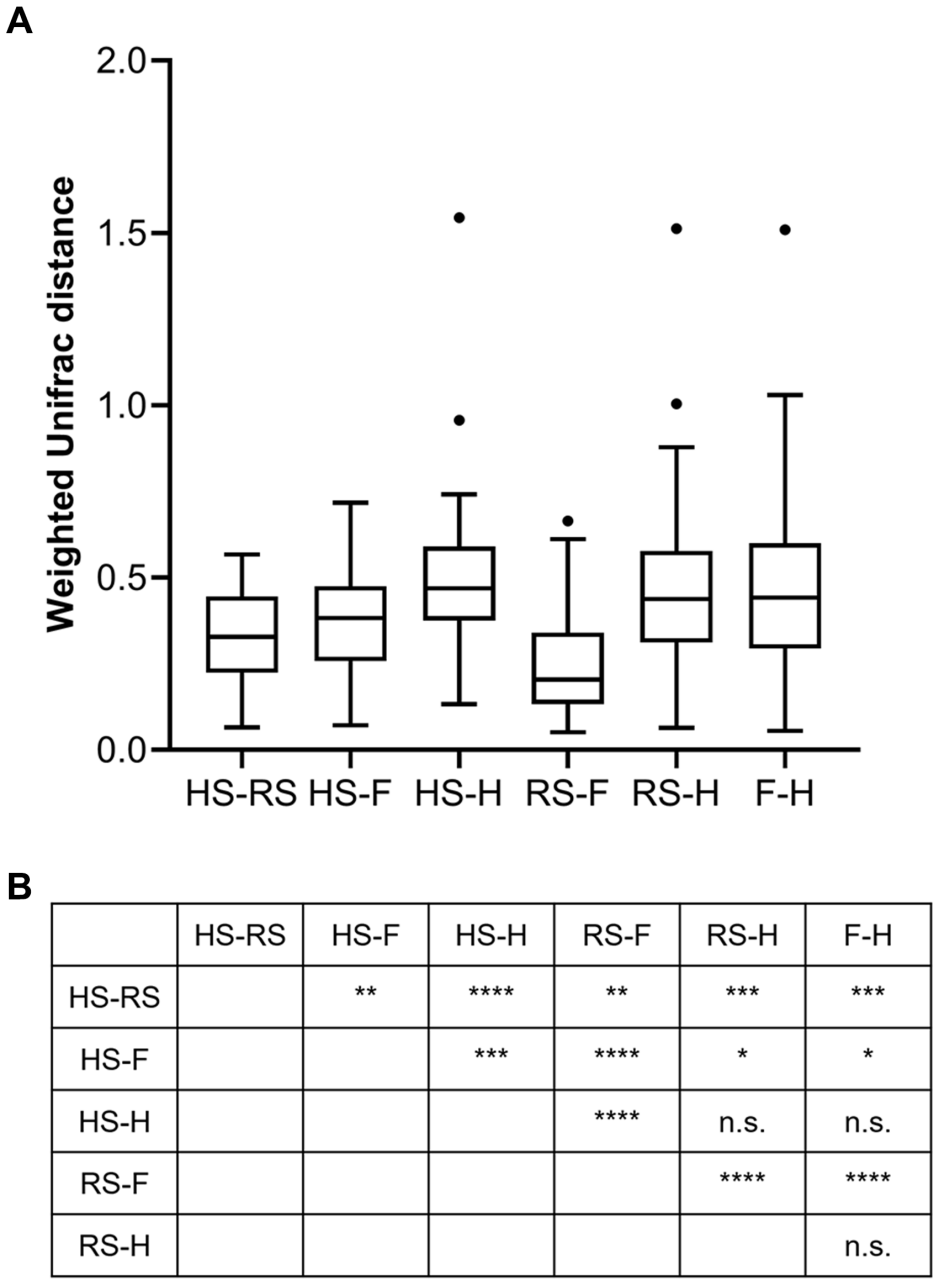
Weighted UniFrac distances between different pairs of sampling sites **(A)** and their significances **(B)**.

An analysis of similarities between microbiomes was performed to explore the relationships among microbiomes from various sampling sites. Weighted UniFrac distances were calculated between each microbiome using a maximum likelihood phylogenetic tree constructed with FastTree. PCoA analysis revealed that the microbiomes at the four sampling sites are significantly different (Figure 4, ANOSIM *p* = 0.001). However, the microbiomes are “paired” in nature, indicating that the four microbiomes from four sampling sites of the same subject form a distinct group of samples. Therefore, simply grouping microbiomes of the same sampling site from different samples is statistically flawed. With this consideration, a second approach was subsequently applied to assess the distance between microbiomes of every pair of sampling sites and to conduct paired *t*-tests to identify significant differences in the distances. Clear differences in distances between each pair of sampling sites can be observed, as shown in Figure 5A. Subsequent statistical comparison (Figure 5A) showed that the distances between the microbiomes of each pair of sampling sites follow a clear hierarchy: hemorrhoid surface and hemorrhoid = rectum surface to hemorrhoid = feces to hemorrhoid > hemorrhoid surface to feces > hemorrhoid surface to rectum surface > rectum surface to feces.

**Figure 6:**
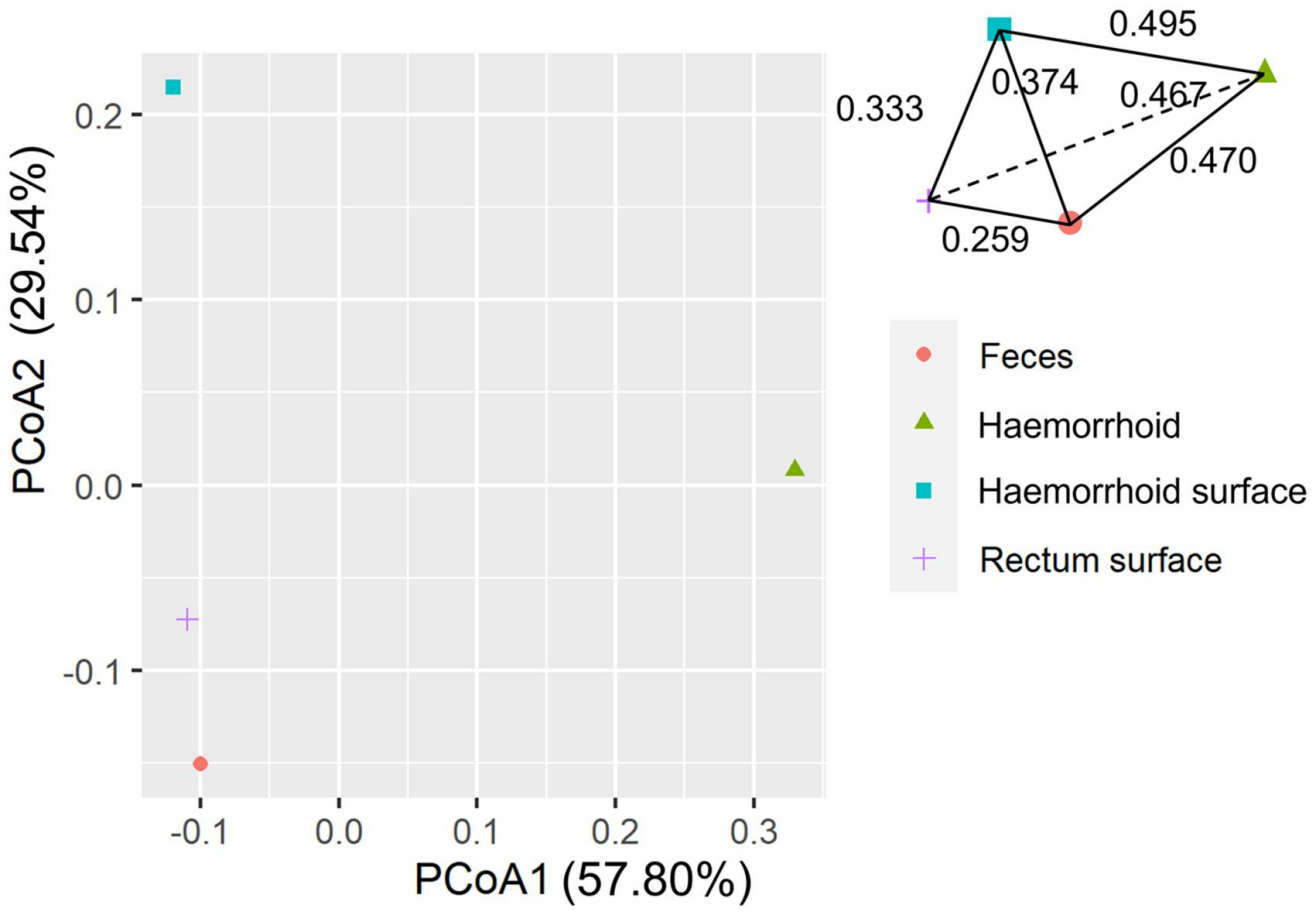
PCoA analysis of microbiomes.

An interesting observation in this distance hierarchy is that the hemorrhoid microbiome is equally distant from other microbiomes, which are closer to each other than to the hemorrhoid microbiome. Although PCoA of averaged distances cannot clearly reflect this observation because it is essentially a dimension reduction technique and cannot accurately represent true distances on a two-dimensional plot (Figure 6), as shown in the three-dimensional illustration of the four microbiomes in Figure 6, it is evident that hemorrhoid microbiomes are distinct from all other three sampling sites. It can therefore be suggested that hemorrhoids harbor a significantly different microbiome compared to all other sampling sites. Considering that all three other sampling sites are part of the gastrointestinal tract and closely interact with the gastrointestinal microbiomes, this finding strongly suggests that hemorrhoid microbiomes may have sources of influence beyond the gastrointestinal microbiomes.

### Bacterial signatures of hemorrhoid microbiomes

To understand the signatures of hemorrhoid microbiomes, a linear model-based MaAsLin2 algorithm was used to identify biomarkers for hemorrhoid microbiomes (Mallick et al., 2021). This method outperforms LEfSe, a similar algorithm, by reducing false positive discoveries. Bacterial genera with an average abundance of over 0.1% and a discovery-adjusted *p*-value (FDR) of <0.05 were reported. No significant microbiomic signature was found in hemorrhoid microbiomes compared to rectum surface microbiomes. However, six genera were found to distinguish the microbiomes of hemorrhoid surfaces, and three genera were found to distinguish the microbiomes of feces compared to hemorrhoid microbiomes. Distinct patterns were found while analyzing the microbiomic signatures of hemorrhoid surface and feces compared to hemorrhoid microbiomes. All six genera identified had higher abundances in hemorrhoid surface samples than in hemorrhoid samples (Table 2), while two out of three bacterial groups identified in the hemorrhoid-feces comparison had higher abundances in hemorrhoid samples (Table 3).

**Table 2:** Microbiomic biomarker of hemorrhoid surface microbiome vs. hemorrhoid.

Taxon	Hemorrhoid surface abundance	Hemorrhoid abundance	FDR
Actinomyces	5.24 × 10^−3^	1.25 × 10^−3^	4.30 × 10^−6^
Corynebacterium	0.018	2.11 × 10^−3^	3.38 × 10^−5^
Finegoldia	0.046	7.03 × 10^−3^	6.19 × 10^−4^
Anaerococcus	0.032	0.010	2.63 × 10^−3^
Peptoniphilus	0.034	0.014	5.23 × 10^−3^
Staphylococcus	0.019	7.68 × 10^−3^	5.23 × 10^−3^

**Table 3:** Microbiomic biomarker of hemorrhoid surface microbiome vs. hemorrhoid.

Taxon	Feces abundance	Hemorrhoid abundance	FDR
Staphylococcus	3.39 × 10^−3^	7.68 × 10^−3^	0.018
Caulobacteraceae	4.64 × 10^−4^	1.67 × 10^−3^	0.018
Bacteroides	0.13	0.076	0.025

The higher abundances of bacterial genera on the surface of hemorrhoids compared to samples from inside the hemorrhoids suggest a bacterial gradient from the surface to the interior of hemorrhoids. This is interestingly true for both feces and rectum surface samples: all six genera are present in significantly higher abundances in hemorrhoid surface samples when analyzed with MaAsLin2. For *Actinomyces,* the average abundances in the rectum surface and feces are 1.11 × 10^−3^ and 1.17 × 10^−3^, respectively, significantly lower than the abundance of 5.24 × 10^−3^ in hemorrhoid surface (FDR = 8.32 × 10^−6^ and 1.33 × 10^−7^, respectively). For *Corynebacterium*, the average abundances in the rectum surface and feces are 2.13 × 10^−3^ and 2.89 × 10^−3^, respectively, significantly lower than the abundance of 0.018 in the hemorrhoid surface (FDR = 2.31 × 10^−5^ and 4.13 × 10^−9^, respectively). For *Finegoldia,* the average abundances in the rectum surface and feces are 0.012 and 5.56 × 10^−3^, respectively, significantly lower than 0.046 in the hemorrhoid surface (FDR = 4.04 × 10^−3^ and 4.29 × 10^−6^, respectively). For *Anaerococcus*, the average abundances in the rectum surface and feces are 9.93 × 10^−3^ and 6.62 × 10^−3^, respectively, significantly lower than the abundance of 0.032 on the hemorrhoid surface (FDR = 9.55 × 10^−3^ and 1.12 × 10^−5^, respectively). For *Peptoniphilus,* the average abundances in the rectum surface and feces are 0.011 and 0.011, respectively, which are significantly lower than the abundance of 0.034 in the hemorrhoid surface (FDR = 0.011 and 1.63 × 10^−4^, respectively). For *Staphylococcus,* the average abundances in the rectum surface and feces are 2.18 × 10^−3^ and 3.39 × 10^−3^, respectively, significantly lower than the abundance of 0.019 on the hemorrhoid surface (FDR = 1.29 × 10^−5^ and 1.63 × 10^−4^, respectively). *Staphylococcus* is a genus particularly associated with the skin and mucosa. The high presence of microbiomes on hemorrhoid surfaces suggests that they originate from the skin.

The hemorrhoid microbiome differs from the fecal microbiome in three genera, including *Staphylococcus*. A higher abundance of *Staphylococcus* was found in hemorrhoids (7.68 × 10^−3^) compared to feces (3.39 × 10^−3^) and the rectum surface (2.18 × 10^−3^). This, in combination with the higher presence of *Staphylococcus* in hemorrhoid samples and the skin origin of this group, appears to indicate a skin origin of *Staphylococcus* in hemorrhoid. Although not studied in this work, previous attempts to analyze fecal microbiomes of healthy adults (Feng et al., 2023; Liu et al., 2023) and children (Yuan et al., 2023) were unable to identify characteristic skin bacteria in their feces. Therefore, a proposal can be made that skin microbiomes at least partly influence the microbiomes in hemorrhoids. It must be stressed here that we did not carry out similar comparisons in healthy subjects. Therefore, this proposal was not validated using healthy controls to determine the absence of skin-oriented microbes in them. Hence, conclusions need to be carefully evaluated.

### Microbes in thrombosed hemorrhoid

**Figure 7:**
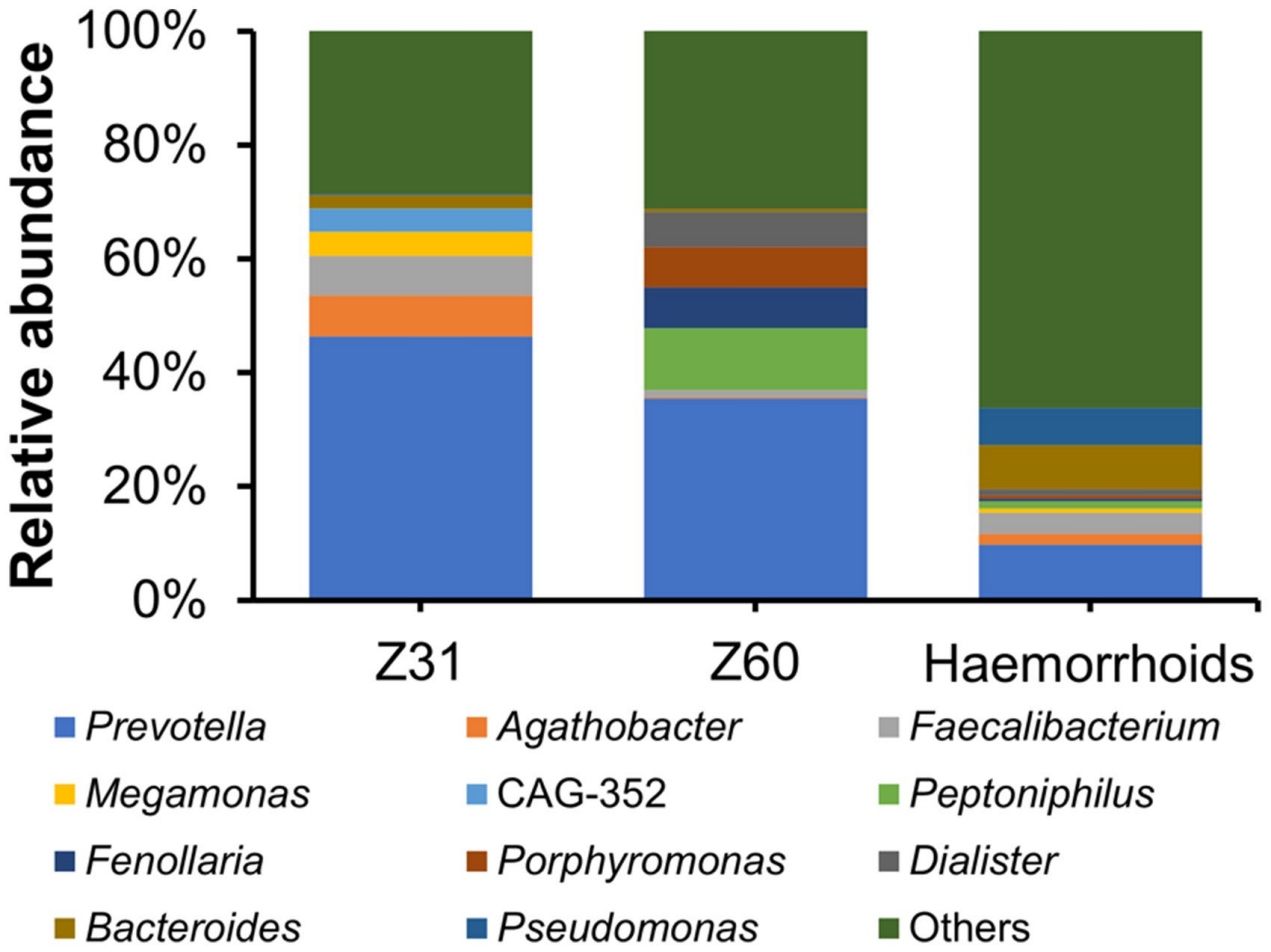
Relative abundance of microbiomes in thrombosed hemorrhoids compared to hemorrhoids.

Two hemorrhoid samples we were able to obtain are from patients suffering from thrombosed hemorrhoids. This is a type of hemorrhoid that can lead to acute pain and swelling (Halverson, 2007). There has been no report or proposal connecting this form of hemorrhoids to microbial infections. The microbiomes associated with the two thrombosed hemorrhoid samples were examined and compared with other hemorrhoid samples. Although the microbiomes in thrombosed hemorrhoid samples Z31 and Z60 appear vastly different from averaged hemorrhoid samples, the most striking observation is that both thrombosed hemorrhoid samples contain a very high level of *Prevotella* (Figure 7; Z31, 46.37%; Z60, 35.45%; averaged hemorrhoid samples, 9.80%). Due to the rarity of patients with thrombosed hemorrhoids, we were unable to collect additional samples for a more comprehensive investigation. Nevertheless, this observation of a high *Prevotella* level implies that *Prevotella* may play a role in the development of thrombosed hemorrhoids, and that the blood clots within thrombosed hemorrhoids do contain microbes. Indeed, preliminary screening successfully isolated bacteria from thrombosed hemorrhoids. However, *Prevotella* strains were not cultivated due to our lack of awareness regarding the potential enrichment of this genus. Additionally, we did not utilize media that could enrich this genus or employ anaerobic cultivating conditions.

## Conclusion

In conclusion, the microbiomic signatures of hemorrhoids and related samples were investigated in 60 patients. The microbiomes of hemorrhoids are significantly different from those found on hemorrhoid surfaces, rectum surfaces, and feces. Further analysis and comparison of microbiomic biomarkers suggest that microbiomes in hemorrhoids are influenced significantly by the surfaces of the hemorrhoids. Specifically, *Staphylococcus*, which are bacteria associated with the skin or mucosa, are more abundant on hemorrhoid surfaces, less abundant in hemorrhoids, and even less so on rectum surfaces and fecal samples. This implies a potential transmission of skin microbes into hemorrhoids. Further investigation of thrombosed hemorrhoid samples suggests a higher *Prevotella* content, indicating the presence of microbes in thrombosed hemorrhoids. Therefore, *Prevotella* may play a role in the development of thrombosed hemorrhoids.

## Data availability statement

The datasets presented in this study can be found in online repositories. The names of the repository/repositories and accession number(s) can be found in the article/supplementary material.

## Ethics statement

The studies involving humans were approved by Medical Ethics Committee of Qilu Hospital of Shandong University (Qingdao) under approval number KYLL-2023045. The studies were conducted in accordance with the local legislation and institutional requirements. The participants provided their written informed consent to participate in this study. Written informed consent was obtained from the individual(s) for the publication of any potentially identifiable images or data included in this article.

## Author contributions

YQW: Data curation, Writing – original draft. WYS: Formal analysis, Writing – original draft. ZL: Writing – original draft & Software. YHW: Data curation, Writing – original draft. LL: Writing – original draft. HX: Writing – review & editing. MW: Funding acquisition, Writing – review & editing. WLS: Funding acquisition, Resources, Writing – review & editing.
